# Real-time assessment of temperature sensitivity, pain processing, and pain behavior across the lifespan: Integrating stimulus data, contact force, and subjective ratings

**DOI:** 10.3758/s13428-026-03192-w

**Published:** 2026-10-14

**Authors:** Heinz-Jürgen Steinhoff, Besim Prenaj, Sebastian L. Kübel, Annekatrin Steinhoff

**Affiliations:** 1 University of Osnabrück, 49069 Osnabrück, Germany; 2 University Hospital of Child and Adolescent Psychiatry and Psychotherapy, University of Bern, Bolligenstrasse 111, 3000 Bern, Switzerland; 3 Graduate School of Health Sciences, University of Bern, Bern, Switzerland

**Keywords:** Heat pain, Cold pain, Pain threshold, Pain-related, Approach and avoidance, Pain intensity, Heat plate, Coldplate

## Abstract

Assessments of temperature sensitivity and pain processing play an important role in psychological and psychiatry research, including with adolescent participants and across the lifespan. To ensure the validity and comparability of findings, experimental settings must be well tolerated and easily manageable for participants of all age groups and reliably monitored by the experimenter. These settings should promote reproducible procedures and support computer-assisted data acquisition and analysis to enable robust, efficient scientific evaluation. We present CAREST (Controlling and Recording Experiments in Sensitivity to Temperature), a PsychoPy-based tool for real-time assessment of temperature sensitivity, pain sensation, and pain response behavior via thermal induction. CAREST controls and communicates with a cold/hot plate and provides a touchscreen slider to assess pain sensation on a visual analog scale (VAS). The plate is placed on a platform with load cells to measure hand-plate contact force, providing an objective assessment of thermal contact between hand and plate surface during the experiment and participants’ approach versus avoidance tendencies in response to pain. CAREST continuously and synchronously records timestamped values of plate surface temperature, participants’ VAS ratings, and hand-plate contact force, enabling computation of several established and novel measures of pain sensitivity and related behavioral factors within a single experimental setting. In a pilot study with 94 participants, we examined heat and cold pain thresholds, intensities, tolerances, and hand-plate contact forces. The results highlight substantial heterogeneity in pain response behavior, with one out of five participants differing markedly from the modest average tendency toward pain avoidance.

**Supplementary Information:**

The online version contains supplementary material available at https://doi.org/10.3758/s13428-026-03192-w.

## Introduction

Assessments of temperature sensitivity and pain processing play a key role in a variety of research endeavors, including in behavioral and psychiatric research. A growing body of research, for example, examines pain sensitivity as a biological mechanism potentially linked with stress-related psychopathology, such as depression (Strigo et al., 2008) and borderline personality disorder (Schmahl & Baumgärtner, 2015), and associated harmful behaviors (e.g., risk-taking and direct self-injury) (Pontén et al., 2025)—a major public health concern (Monto et al., 2018; Patalay & Gage, 2019; Steinhoff et al., 2021).

In experimental human pain testing, thermal stimuli applied to the skin are well-established tools for inducing spontaneous pain (Bakkers et al., 2013; McDaniel et al., 2023; Reddy et al., 2012; Rolke et al., 2006). These methods are particularly valuable for assessing thermal detection, pain thresholds, and pain tolerance. They typically use thermodes, devices that precisely control stimulus parameters such as temperature, timing, and ramp rate. When applied according to standardized thermode-based protocols for quantitative sensory testing (QST), these methods allow for a comprehensive assessment of thermal pain sensitivity and related parameters (Bakkers et al., 2013; Rolke et al., 2006) important, e.g., to identify an individual’s neuronal pain mechanisms and evaluate or predict responsiveness to medications (Georgopoulos et al., 2019; McDaniel et al., 2023; Weaver et al., 2021). However, to facilitate integrative analyses of neuronal, physiological, cognitive, and behavioral factors in response to pain, a tool is needed that provides continuous, high-resolution, and timestamped data recordings of pain stimuli (e.g., temperatures), pain onset, its perception (e.g., pain intensity), and pain behavior (e.g., approach versus avoidance), thereby enabling temporal alignment with concurrently acquired physiological signals (e.g., ECG, EEG) and a multimodal understanding of pain.

Psychopathology that has been associated with altered pain processing, such as depression and borderline personality symptomatology, as well as risk-taking behaviors and self-injury, typically emerge during the first decades of life and then vary across the lifespan (Steinhoff et al., 2021; Videler et al., 2019). Accordingly, assessments aimed at a comprehensive understanding of pain-related mechanisms require methodologies that are applicable and comparable across different life stages, including childhood. Conducting pain studies with children presents unique ethical and methodological challenges. Foremost among these is the duty to protect child participants by ensuring a favorable risk–benefit balance and safeguarding their well-being throughout the research process. Researchers must guarantee that no painful stimulus exceeds the child’s tolerance limit and must provide immediate means for the child to stop or withdraw from the experiment at any time (Charlton, 1995). Although many pain-inducing devices used in QST are hardware-limited to safe levels and do not pose a risk of tissue damage (Rolke et al., 2006), it may be nonetheless essential to tailor stimulus intensity to the child’s subjective perception of mild or moderate pain and to allow the stimulus to be terminated before reaching high levels of discomfort or pain tolerance (McDaniel et al., 2023). Additionally, methodological adaptations are necessary to account for children’s limited attention spans, which can compromise the reliability of pain threshold estimation (Manning et al., 2018).

Here, we build on and advance the application of a hot plate that has been used in prior psychological and psychiatric pain studies with adolescents (Kao et al., 2024; van der Venne et al., 2021) and meet the above requirements. In contrast to well-established stimulators such as CHEPS (Contact Heat Evoked Potential Stimulator) or ATS (Advanced Thermal Stimulator) (Bakkers et al., 2013; Chen et al., 2001; Rolke et al., 2006), a heat plate mimics a real-world scenario like putting a hand on a warm surface. Heat plates, as well as cold plates—which have not been used in the above-cited studies—do not need to be affixed to a specific body region as recommended for the smaller thermodes (Bakkers et al., 2013; Pavlaković & Petzke, 2010). This reduces setup time and potential discomfort or anxiety, especially for young participants, because they can withdraw from the plate and thus easily stop the experiment at any time. The plate’s surface is large enough to heat or cool, e.g., the entire palm, independent of its size, which avoids biases related to different thermode/palm area size ratios due to different body sizes that result in different spatial summation effects. This is especially relevant if the aim is to study and compare pain processing across age groups from a lifespan perspective. A cold/heat plate helps keep potential skin temperature stress as low as possible, as the method of limits and a larger stimulated skin area reliably lower the heat pain threshold (Park et al., 2019; Rasmussen et al., 2015). This effect is supported by low rates of temperature increase or decrease, which lead to significantly higher pain ratings (Vierck et al., 2013) and reduce the impact of participants’ reaction times, thus making experiments practicable for children.

Beyond previous applications of heat plates in psychological and psychiatric research, we equipped the cold/hot plate with force sensors to measure hand contact force on the plate surface, offering a more comprehensive view of cold/heat pain processing. Potential individual variations in contact force may modulate the contact area between the participant’s hand and the plate surface, as well as the quality of thermal connection, and thus the number of receptors in contact with the plate and the rate of thermal transfer (Defrin et al., 2009). Both parameters may determine the onset and intensity of pain sensation (Defrin et al., 2009). The force sensors not only facilitate experiments under standardized hand contact force conditions but also enable monitoring of respective values during the experiment—which can be made visible or invisible to the participants, depending on a specific research question. The corresponding data can subsequently be correlated with self-reported thermal and pain sensations (Maeda & Sakakibara, 2002). We argue that, especially if the force sensors are invisible to participants, recordings of hand-plate contact force enable assessment of automatic motor and behavioral responses indicating thermal preference or avoidance, e.g., latency to remove from the stimulus. For example, it may be of interest to examine interindividual differences in participants’ motor and behavioral responses to an initial sensation of pain and increasing pain intensity. One may also ask whether some individuals respond with increasing or decreasing contact force before they reach their tolerance values, and whether these patterns reflect, for instance, rising muscular tension versus flight responses or hesitation.

We have integrated a time-synchronous visual analog scale (VAS) (Huskisson, 1974) for continuous self-reporting of pain sensation on a touch screen monitor. Pain rating using a VAS was found to be a valid and reliable measure for assessing pain, including in children (von Baeyer & Spagrud, 2007), and applicable in the context of cold/hot plates (Kao et al., 2024; van der Venne et al., 2021). A continuous VAS rating on the touch screen is unaffected by reaction time and may therefore be particularly suitable for children, given their limited attention spans (Manning et al., 2018). The scale we integrated ranges from 0, “no pain”, to 10, “very severe pain”.

We developed software, CAREST (Controlling and Recording Experiments in Sensitivity to Temperature), based on PsychoPy (Peirce et al., 2019), that enables us to control the cold/hot plate while at the same time continuously recording the plate’s surface temperature and the participants’ VAS ratings, along with the hand-plate contact force. In what follows, we show how to employ the tool and derive relevant measures of pain sensation (e.g., pain threshold, intensity, endurance, tolerance, and contact force). We present reference data from 94 adult participants who completed heat and cold pain experiments in a standardized fashion. We show how hand contact force can measure individual pain approach and avoidance. Finally, we discuss how users can adapt the tool for individual research purposes, for example, to contribute to a better understanding of pain physiology in child, adolescent, and adult psychopathology.

### Participant setup

**Fig. 1:**
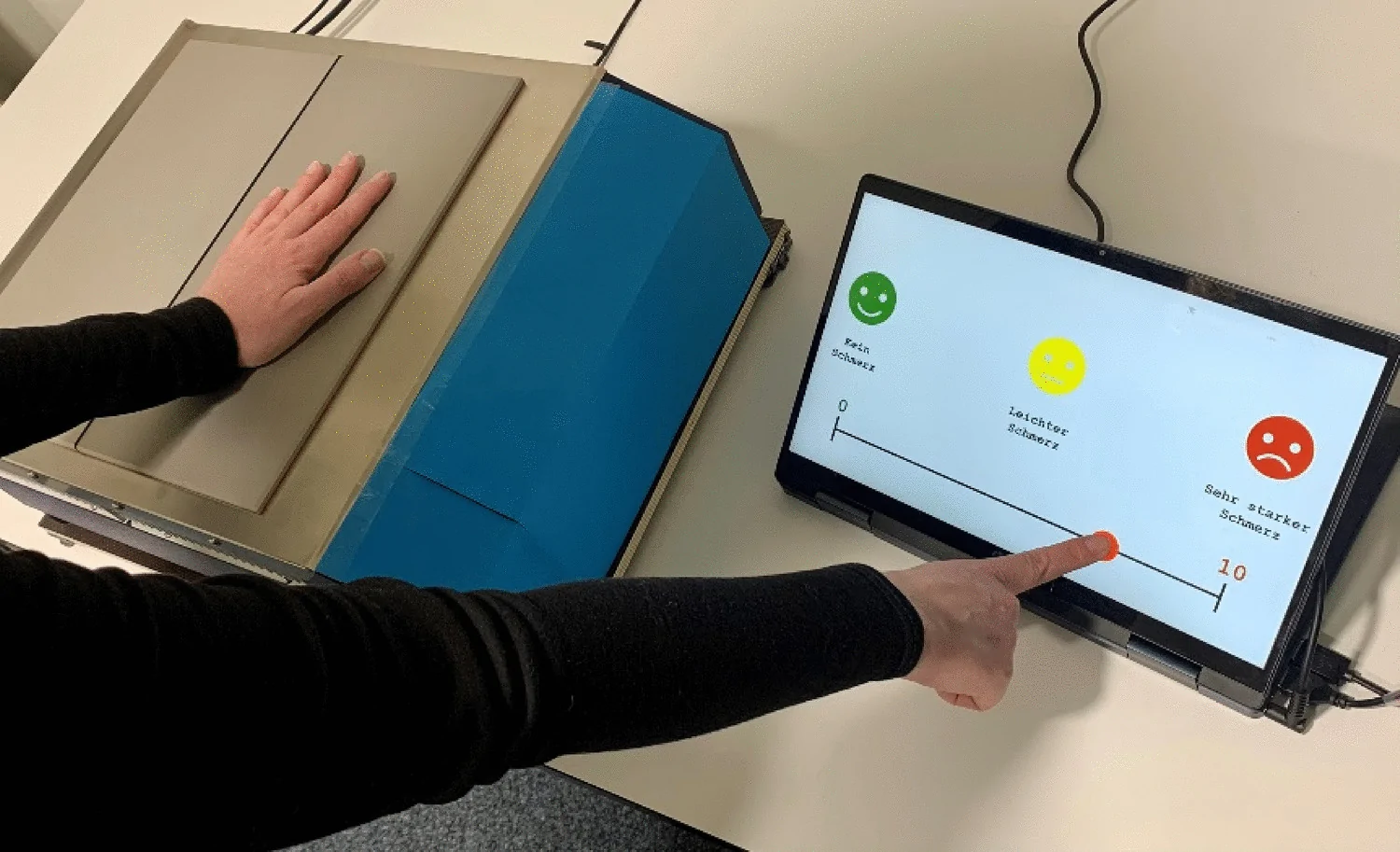
Experimental setup for participants: cold/hot plate placed on a platform supported by a scale with four load cells and a touch screen displaying the visual analog scale and slider

Figure 1 shows the experimental setup using a dual cold/hot plate (AHP-1200, Thermoelectric Cooling America Corporation, Chicago, IL, USA) placed on a platform with four load cells (scale). To avoid potential bias from participants observing the temperature display during the experiment, the plate’s screen showing the target and actual temperatures was covered.

At the start of CAREST, after entering participant and session numbers, the system heats or cools the plate surface to the predefined baseline temperature: 35 °C for heat pain and 25 °C for cold pain experiments. A standby screen remains visible until the participant uses a slider on the touch screen to tare the scales immediately before the experiment begins. The participant should now place their non-dominant hand on the active plate surface, as this likely facilitates smoother VAS ratings on the touchscreen, which can then be provided using the dominant hand (Kao et al., 2024; van der Venne et al., 2021). Practical considerations, particularly in studies involving children, include adapting to handedness (e.g., with a rotatable) and ensuring ergonomic hand positioning through a height-adjustable table or footstool.

For our data collection, we maintain the baseline temperature for three minutes to allow the participant’s hand temperature to adapt while the experimenter delivers standardized experiment instructions (e.g., an explanation of the procedure) tailored to this duration. This procedure is expected to minimize participant fatigue during the adaptation phase. The phase duration can be adjusted based on the research design and environmental conditions. For example, room temperature or season may influence participants’ hand temperature before the experiment begins and should thus be considered when planning the duration of the adaptation phase. After this phase, a linear temperature ramp starts. In our setup, the plate is either heated up to a peak temperature of 47.4 °C within 156 s (13 s/°C, corresponding to a rate of 0.08 °C/s) (heat pain experiment) or cooled down to 5 °C within 280 s (14 s/°C, corresponding to a rate of 0.07 °C/s) (cold pain experiment).

Our standardized experiment instructions to be delivered during the adaptation phase include that participants can familiarize themselves with the VAS rating procedure and assess hypothetical pain intensity based on three vignettes of painful situations. Researchers frequently use hypothetical pain vignettes to train participants in using pain intensity rating scales (Breau et al., 2001; Jaaniste, Noel, & von Baeyer, 2016). We suggest that these assessments can be used to check a) whether participants understand how to use the touch screen slider; b) whether they understand the structure of the VAS and can use it to differentiate between non-painful and painful experiences. Pain ratings for hypothetically painful situations are expected to exceed values indicating at least mild pain (i.e., values ≥ 50). Additionally, this procedure may be suitable for identifying c) individual differences in how participants understand and use the VAS, for example, regarding the degree of differentiation (e.g., spacing) between ratings of various painful experiences—however, such use of hypothetical vignettes requires a level of standardization and validation (e.g., randomized order of assessment) that has not been the focus of our present investigations.

In our case, we used three hypothetical situations, of which at least one should be understood as not or only mildly painful, whereas at least another one should be understood as clearly painful (i.e., a VAS rating ≥ 50 is expected). Specifically, we ask participants to rate the intensity of pain from a) stumbling and falling on the snow, b) stumbling and falling on the pavement when there is no snow and scraping the knee, and c) pinching a finger in a car door (Breau et al., 2001). It is worth noting that responses may, to some extent, reflect participants’ real and subjective past experiences and respective recollection of pain in situations like those described in the vignettes.

During the remainder of the adaptation phase and the following heating/cooling phase, participants can continuously rate pain intensity using the touch screen slider. As was done in previous research using heat plates (Kao et al., 2024; van der Venne et al., 2021), we instruct the participants to put their hand firmly on the plate and withdraw it to stop the experiment as soon as they perceive pain as intolerable. During the rating phase, we gave no additional instructions to allow observation of participants’ natural contact force dynamics in response to changing temperatures and pain.

**Fig. 2:**
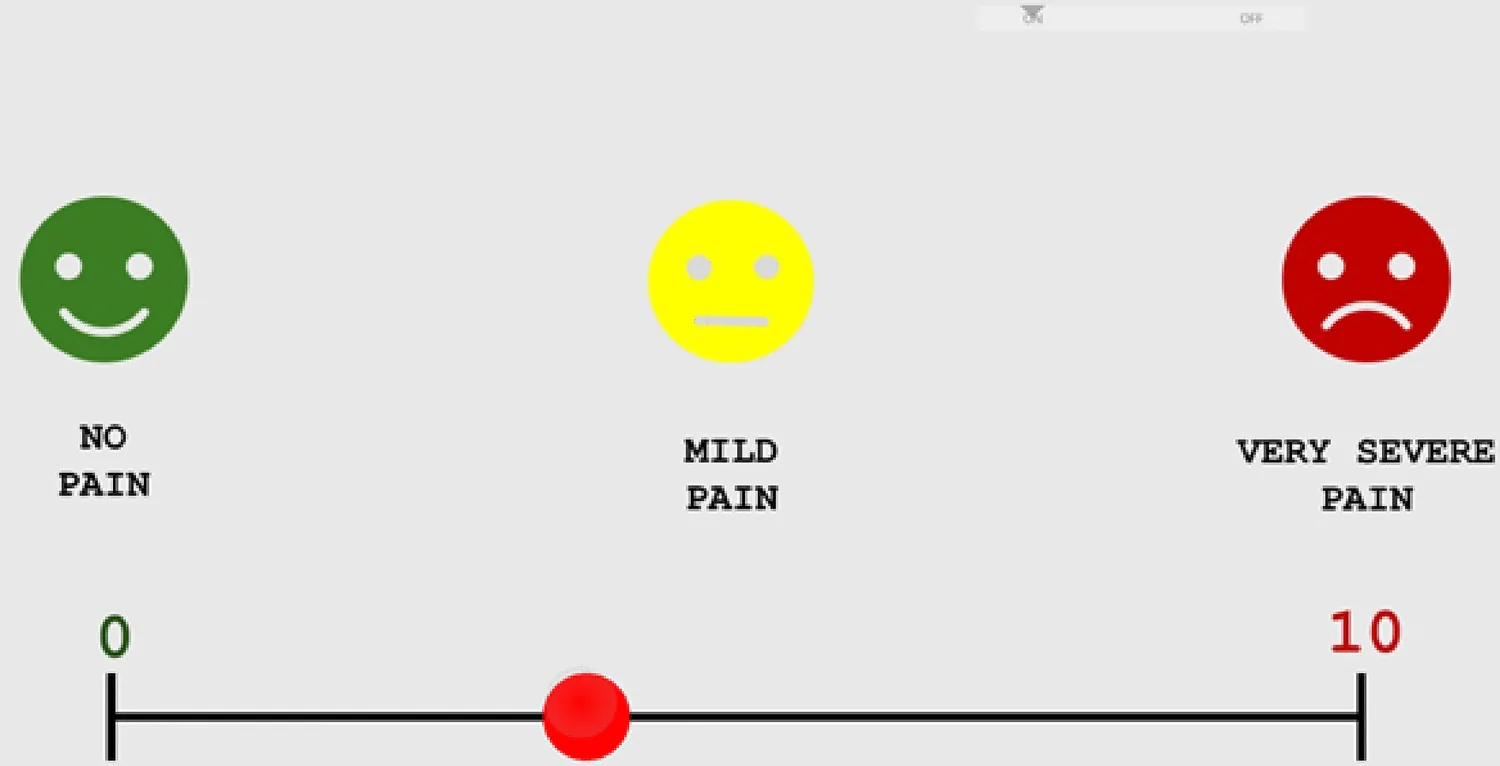
CAREST’s default visual analog scale image as exchangeable background with touch screen slider for pain sensitivity testing. The snapshot shows the rating slider (*red dot*) shifted towards the “mild pain” cut point

Pain measurement using classic unidimensional scales such as the VAS, the Verbal Rating Scale (VRS), and the Emoji Faces Pain Scale (Emoji-FPS) has demonstrated both reliability and validity, including in pediatric and adolescent populations (Bakkers et al., 2013; Li et al., 2023; Scott & Huskisson, 1976). However, accurately classifying VAS scores has proven challenging (Boonstra et al., 2014; Hirschfeld & Zernikow, 2013; Serlin et al., 1995), prompting recommendations for the use of predefined cut points to enhance consistency and comparability across studies and interpretability of findings (Hirschfeld & Zernikow, 2013). In our current study, we assess pain intensity using a VAS ranging from 0 to 10, computed on a 0 to 100 scale within the software. The scale includes verbal and visual anchors: “no pain” at the lower end, “mild pain” at the midpoint, and “very severe pain” at the upper end (Fig. 2). These anchors are reinforced by graphical representations using three Emojis (smiling, neutral, and frowning faces, respectively). This configuration effectively positions “mild pain” at the midpoint of the scale, framing the present VAS as a zoomed-in version of a full-range VAS that typically extends to “worst imaginable pain” (Nielsen et al., 2009). Our modified scale allows more fine-grained assessment of low- to moderate-pain intensities, aligning with the design of the present experiment, which is intended to avoid exposing participants to extreme pain levels. The milder anchor descriptor may also be useful in experiments, especially with children, to avoid intimidation, and researchers using CAREST can choose any other descriptor they deem suitable for the specific participant (e.g., age-) groups they are studying.

CAREST users can replace the background image with other VAS images as needed—for example, to expand or compress the scale by using different labels for the minimum and maximum categories (e.g., “worst imaginable pain”). In some previous studies, experimenter instructions specified that participants should start rating pain intensity on a VAS only after the very first perception of pain (i.e., at their pain threshold) (van der Venne et al., 2021). For our study, however, we instructed participants to begin rating their pain perception at the start of the experiment, allowing VAS ratings that indicate “no pain.” During the adaptation phase, when the temperature is held at a pleasant level, this rating provides the baseline for determining the pain threshold during the following temperature increase or decrease (see below). This approach is intended to ensure that participants with low pain tolerance do not feel pressured to continue the experiment for social reasons—for example, out of a belief that the actual task (i.e., rating) does not begin until pain is clearly felt, and that they should wait to do it “correctly.” In addition, continuous rating throughout the experiment makes analysis less dependent on individual reaction time and attention span (Manning et al., 2018). In this setting, we also aim to capture individual differences in behavioral tendencies to approach or avoid potentially painful situations. Avoidance, for instance, may be reflected in participants reducing hand-plate contact force or terminating the experiment before or shortly after the onset of pain.

We monitor the hand-plate contact force during the entire experiment by reading out the load cells synchronously to the values of the VAS pain rating and plate surface temperature. The hand-plate contact force is displayed, with color coding (green for contact force > 2 N, which can be altered in the software if needed), on a small screen that we installed on the platform. This can be covered or hidden during the experiment, for instance, to generate objective measures of participants’ tendencies to approach the stimulus —indicated by a rise in the contact force—or to avoid it—indicated by a lowering of the contact force. However, the experimenter and/or participant may also examine the screen during the experiment if the aim is to ensure that all participants apply an identical contact force, thereby minimizing variability in thermal transfer and maximizing the comparability of pain sensitivity measures (e.g., threshold, intensity, and tolerance). The final drop of the contact force to 0 allows for precise documentation of the participant’s hand withdrawal independent of the VAS rating that might still be ongoing.

If the participant does not stop earlier, the experiment ends when the plate reaches its predefined peak temperature. The plate surface temperature is then actively brought back to the programmed baseline temperature.

### Measures derived from CAREST data

Data generated using CAREST can be used to measure several concepts relevant to temperature sensing and pain research (Table 1). The concepts and measures are based on (continuous) recordings of time, temperature, VAS ratings, contact force, and combinations of these parameters. In the following, we outline how researchers can utilize the data to capture both established concepts—such as pain intensity, pain threshold, and pain tolerance (see, e.g., Bakkers et al., 2013; Rolke et al., 2006) —and novel concepts that have not yet been operationalized in prior research using heat or cold plates, such as hand-plate contact force.

**Table 1:** Key concepts and measures that can be extracted from or computed based on the data provided by CAREST. For established measures, see, e.g., Bakkers et al. (2013) and Rolke et al. (2006)

Concepts	Measures^a^
Temperature perception	Temperature deviance from baseline at which participant detects warming/cooling (e.g., using a VAS or categorical options)
Temperature tolerance (censored temperature tolerance)	Maximum/minimum temperature perceived as tolerable; temperature at which participant withdraws hand, in experiments with increasing stimulus (cooling or heating); time until participant withdraws hand or stops the experiment in experiments with constant temperature. The inherent limitation of heat and cold pain experiments due to safety reasons is accounted for by referring to the resulting estimates as *censored temperature tolerance*
Pain intensity	VAS rating during the experiment, at specific temperatures, e.g., at noxious temperatures (> 44 °C, < 15 °C (Dubin & Patapoutian, 2010; Rolke et al., 2006)), or at maximum temperature perceived as tolerable
Pain threshold	Temperature of first pain sensation, here indicated by the first significant increase in VAS pain rating above individual VAS baseline (see text)
Pain endurance	Time between reaching pain threshold and intolerable pain (in experiments with constant temperature set at level typically or individually perceived as painful: overall time until last VAS rating recorded)
Pain tolerance	Maximum pain intensity that the subject is willing to endure (Nielsen et al., 2009)
Tolerated pain load	VAS ratings integrated over time until tolerance in experiments with constant temperature (area under the curve)
Pain intensity dynamics	Trajectory of pain intensity across time/temperature (Potvin et al., 2024)
Hypothetical pain intensity	VAS ratings for hypothetically painful situations (e.g., pinch fingers, scrape knee); provides reference ratings and information on how the VAS is used by individual participants, based on imagination or previous real experiences
Hand-plate contact force	Average and standard deviation of hand-plate contact force (in Newtons); Temperature at which contact force falls to zero determines intolerable pain (termination of experiment)
Contact force dynamics	Trajectory of hand-plate contact force across time/temperature
Relative contact force change (pre/post threshold) as measure of behavior: approach vs avoidance	Intra-individual variation of contact force in response to variation of the stimulus: difference of the averaged contact forces in the temperature intervals above and below pain thresholds divided by the contact force averaged over the entire experiment

^a^Experimenter instructs that participants withdraw hands “when intolerable pain is reached.”

*Pain intensity* ratings can be compared within and between participants for any given temperature, e.g., to compare how intense the typical threshold of noxious pain is perceived by different participants. In turn, one can identify temperatures at intensity ratings of specific interest, such as the maximum pain intensity that a participant is willing to endure (Kao et al., 2024; van der Venne et al., 2021).

To assess the *pain threshold* using VAS ratings, participants in the pilot study reported below were instructed to rate their perceived pain intensity starting from the initial stimulus adaptation phase. Ideally, VAS ratings remain at 0 (“no pain”) during this phase and increase only upon the first perception of pain. However, retrospective VAS ratings for pain intensity at heat and cold pain thresholds across various methods were reported to range between 20/100 and 50/100 (Kelly et al., 2005; Kuhtz-Buschbeck et al., 2010; Wasner & Brock, 2008), indicating that even the lowest reported intensities at threshold are considerably higher than the scale minimum (e.g., 1/100). In addition, in the present synchronous VAS recording, low-level fluctuations occur at the lower end of the scale. These may reflect responses to pre-threshold sensations—such as non-painful thermal discomfort—as well as non-perceptual activity and touchscreen input variability resulting from initial finger placement. To account for these factors, baseline VAS ratings were determined during neutral temperature intervals (36–37 °C for heat and 23–24 °C for cold pain experiments).

Given that the transition from non-painful to painful sensation is nonlinear and occurs over a narrow range of stimulus intensities (Johnstone et al., 2012), we define the pain threshold as the temperature at which the VAS rating first increases significantly above baseline. Specifically, we calculated this value as two standard deviations plus a constant of 10, the latter of which accounts for finger placement and movement variability. We propose that this inflection point in the VAS curve represents the psychological transition to a painful sensation. This approach distinguishes the perceptual onset of pain from the cognitive labeling of this experience, the latter of which may occur at higher temperatures as participants may hesitate to categorize a sensation as “pain” until they are certain (Kuhtz-Buschbeck et al., 2010).

As an additional measure, or in cases where the inflection point is not statistically significant, we employ a categorical (mild) pain threshold, defined as the point where the VAS rating exceeds the ‘mild pain’ anchor (VAS 50). However, it should be noted that relying on a fixed VAS value or a specific pain category to define the threshold may introduce additional variance, as participants may vary in their subjective interpretation of pain categories, qualitative anchors, or the application of the VAS scale. In the pilot study presented below, we show data for both measures. Because the rate of temperature change can influence the pain threshold (Bakkers et al., 2013), identical ramp rates and baseline temperatures must be maintained for intra-sample comparisons.

Measurement of *pain endurance* requires computing the time between a participant’s first pain sensation and hand removal (i.e., termination of the experiment). *Pain tolerance* (i.e., how much pain a participant tolerates) and *temperature tolerance* (i.e., maximum [heat experiment] or minimum [cold experiment] temperature tolerated) are determined using the maximum pain intensity rating and the maximum/minimum temperature, respectively, that are reached during the experiment (Nielsen et al., 2009; van der Venne et al., 2021).

In addition, we suggest assessing what we call *tolerated pain load*. This measure integrates pain intensity ratings until tolerance in experiments with constant temperature. Measuring tolerated pain load enables the assessment of inter-individual differences in subjective pain perception without prioritizing any single parameter, such as temperature, duration, or intensity, when comparing pain tolerance. For safety reasons, we terminate the procedure once temperatures of 5 °C (cold plate) or 47 °C (heat plate) are reached. These predefined upper and lower limits may fall below or above some participant’ actual tolerance thresholds, preventing accurate determination of their true heat or cold pain tolerance and reducing discrimination at the upper end of the measurement range (Kao et al., 2024; van der Venne et al., 2021). We account for this inherent limitation of heat and cold pain experiments by referring to the resulting estimates as *censored temperature tolerance*.

Tolerated pain load may offer meaningful new insights by allowing researchers to categorize participants based on their willingness to endure pain. With its exact and high-resolution recordings of temperature and corresponding intensity ratings, the data generated by CAREST is also suitable to explore *pain intensity dynamics* (i.e., trajectories) using complex statistical modeling (Potvin et al., 2024).

*Hypothetical pain intensity,* based on the hypothetical pain vignettes, can be assessed using the same rating scale, before or after the main experiment. These ratings indicate how participants use the VAS scale for self-ratings and provide a reference for interpreting pain intensity associated with different temperatures compared with real-life situations.

The average value of a participant’s *hand-plate contact force* may be used to adjust individual differences in other measures (e.g., pain endurance and tolerance) for variations in thermal transfer, thereby reducing bias in statistical modeling. Contact force at specific points during the experiment provides further insights: for example, contact force reaching zero is a reliable measure of hand removal upon reaching the individual point of intolerable pain (i.e., termination of the experiment). *Contact force dynamics* (e.g., the progression of the hand-plate contact force in response to rising or decreasing temperatures) may reflect physiological reactions (e.g., muscular tension) to pain sensation or behavioral tendencies to avoid or actively engage with the stimulus. Specifically, approach versus avoidance may be quantified by the relative change of the contact force from before to after reaching one’s pain threshold. Other interpretations of contact force dynamics are also possible, for example, participant fatigue.

**Fig. 3:**
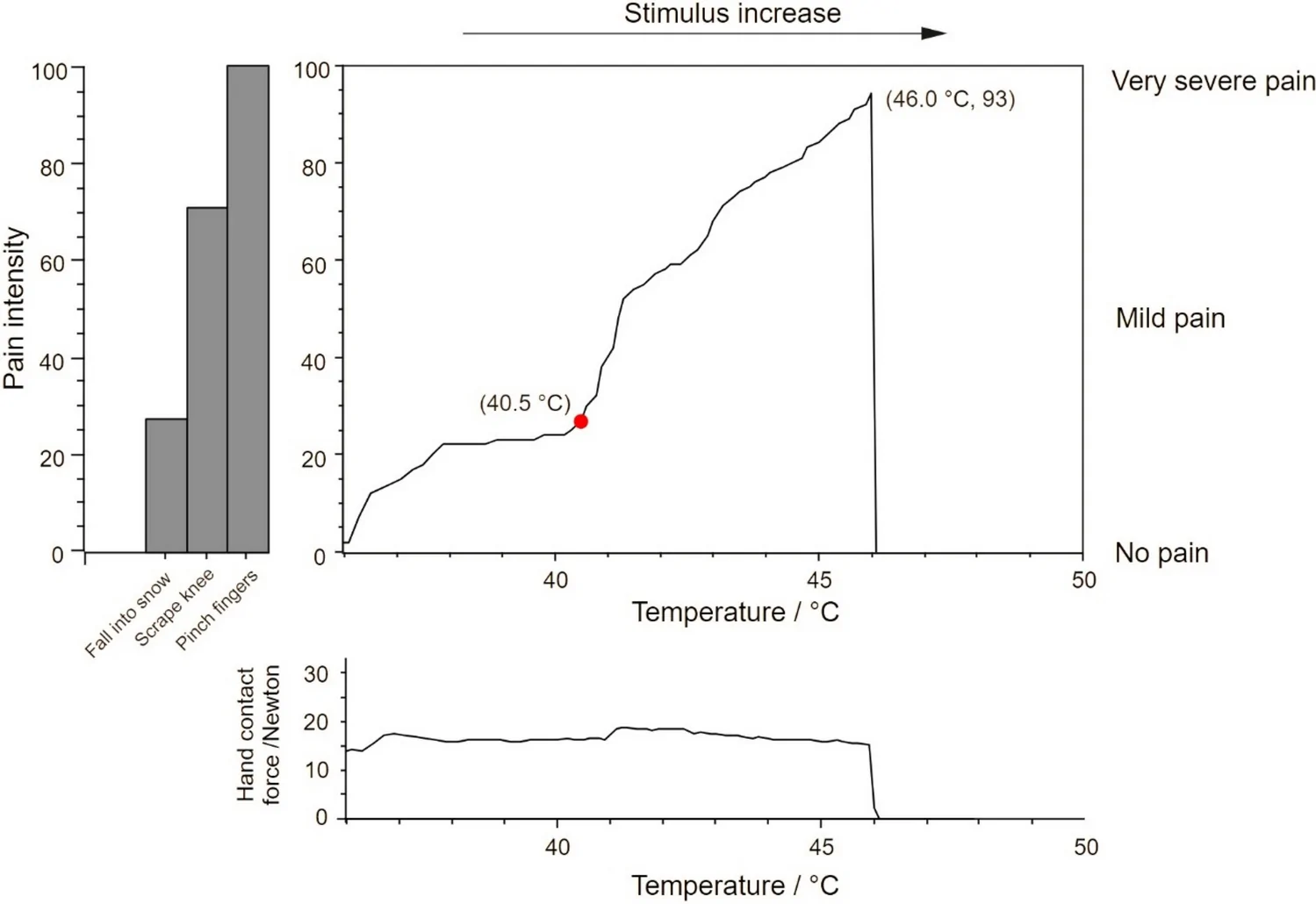
Different measures provided by the heat pain experiment. The bar graph shows pain intensity self-ratings for three imagined pain stimuli; the line graph shows self-ratings vs. temperature for determination of pain threshold (*red dot*, 40.5 °C), pain tolerance (93), and temperature (at pain) tolerance (46.0 °C), i.e., the last self-rating and temperature before the participant’s hand was withdrawn as indicated by the sharp decrease of the hand contact force to zero

Figure 3 illustrates data of a heat pain sensitivity experiment performed by one of the authors using CAREST (the raw data are available from GitHub). The self-ratings of pain intensity associated with three hypothetically painful situations displayed on the left hand-side show an increase with the imagined increased risk of injury. The pain intensity ratings for these three situations show how the participant used the full range of the scale, differentiating between a painless incident (27), associated with falling into soft snow, and pain intensity close to “very severe pain” (100) associated with pinching fingers. This rating behavior may demonstrate the ability to differentiate between diverse levels of pain and use the self-rating scale accordingly.

In the heat pain test shown  in Fig. 3, the person’s pain threshold was reached at 40.5 °C, as indicated by the following significant pain intensity increase above baseline. Mild pain was perceived to be just above 41 °C, and intolerable pain was reached at 46.0 °C. The latter was associated with the participant’s maximum pain intensity rating (93), close to “very severe pain” (100), which was similar to the rating associated with the hypothetical situation of pinching fingers. Pain was tolerated over the course of 72 s with a temperature increase of 5.5 °C. VAS rating was stopped when the hand was withdrawn from the plate. The hand-plate contact force trajectory displays a nearly constant contact force before the hand is withdrawn at 46.0 °C.

### Hardware and software setup

CAREST guides the communication between the experimenter and the participant via control and rating sliders on a touchscreen laptop, and controls the cold/heat plate and scales hardware via USB ports. The following describes the main hardware and software components. The software package is openly available from https://github.com/hjuergensteinhoff/CAREST.

### Hardware

The experimental setup consists of an AHP-1200 dual cold/hot plate (Thermoelectric Cooling America Corporation, Chicago, IL, USA) placed on a scale platform with four load cells (M5Stack Technology Co., Ltd., China). An HX711 24-bit A/D converter chip (M5Stack) digitizes the load-cell analog signals and transmits them to a touchscreen laptop (Windows 11) via an ESP32 Basic Core IoT Development Kit V2.7 (M5Stack) connected through USB. The internal temperature controller (ATEC302) of the AHP-1200 cold/hot plate is connected to the second USB port of the laptop, enabling data transfer for programming the controller and recording the plate’s temperature. Following hardware installation, the load cells were calibrated using known weights ranging from 10 to 50 N, achieving a measurement error of less than 1% within this interval. For forces below 10 N, the error is determined by noise (peak-to-peak noise amplitude ~ 0.1 N), while for forces exceeding 50 N, non-linearity increases the error up to 1.6% at 100 N.

### Software

The ESP32-based scale interface runs the program ‘scale.ino’. This program reads the signals from the four load cells via an HX711 A/D converter at a rate of ten samples per second. After applying the calibration factor, the calculated force values are stored, displayed on the M5Stack LCD, and transmitted on request from CAREST via the serial connection. Additionally, a color-coded background on the display visually indicates whether the measured force is below (red) or above (green) a defined threshold. The scale can be forced to tare by sending “0” to its serial port. Serial communication between scale.ino and CAREST is prompted by a blinking dot in the lower-left corner of the LCD display.

CAREST.psyexp was developed using the PsychoPy software package (Peirce et al., 2019) using Code components for interaction with the plate and scale. The “library” “HP_commands_config.py” contains the code for controlling and communicating with the ATEC302 temperature controller (commands were coded according to command byte tables published in the ATEC302 TE Temperature Controller Reference Manual Rev 1.10, Mar, 2018, received from Thermoelectric Cooling America Corporation). It reads the user-editable input file “config.dat,” which contains the temperature and timing values for the cold or heat pain experiments. Table 2 shows a sample config.dat file for a heat pain experiment, as described in the Participant Setup section. For safety reasons, baseline temperatures are limited to values in the range between 10 °C and 45 °C, peak temperatures are limited to values between 5 °C and 49 °C. The ramp-up rate to the peak temperature is limited to less than 1 °C/13 s, and the plate is actively cooled upon reaching the peak temperature (hold_time_pt = 0) to avoid non-acceptable overshooting of the target temperature. The same rate was used in published studies using a similar hot plate (Kao et al., 2024; van der Venne et al., 2021). With our default configuration (Table 2), the maximum plate-surface temperature reaches 47.4 °C before active cooling brings it below 47 °C in less than 20 s. The small overshooting of the peak temperature by 0.4 K is due to the present PID controller settings (factory defaults), which are a compromise enabling fast heating and avoiding large peak temperature overshooting. The dwell time above 47 °C (20 s) is mainly given by the plate’s heat capacity and the plate’s cooling performance.

**Table 2:** Config.dat example file for heat pain experiments

# Configuration File for Temperature and Timing Settings	
# Format: key = value	
# Please keep the key names and use one decimal place for temperatures and whole numbers for time in seconds	
# –- Port Settings –-	
serial_port_HP = COM3	# COM port for controlling the heat plate
serial_port_scales = COM6	# COM port for controlling the scales
# –- Temperature Settings (in Celsius) –-	
base_temperature = 35.0	# Baseline temperature before start of experiment and during adaptation #(limits:10.0—45.0)
peak_temperature = 47.0	# Peak temperature reached during the experiment (limits: 5.0—49.0)
# –- Timing Settings (in seconds) –-	
ramp_up_time_bt = 60	# Time to reach baseline temperature if not reached before start of experiment
hold_time_bt = 120	# Time to hold at baseline temperature (adaptation)
ramp_up_time_pt = 156	# Time to reach peak temperature (limit: > 13 s/C)
hold_time_pt = 0	# Time to hold at peak temperature
ramp_down_time_bt = 300	# Time to reach baseline temperature after peak

For a cold pain experiment the cooling rate from baseline (25 °C) to peak temperature (5 °C) was set to 1 °C/14 s as default. For temperatures below ~ 8 °C, the measured maximum cooling rate of the present plate approaches 1 °C/18 s because of its limited cooling power. Therefore, a hold time at peak temperature (hold_time_pt = 60) guarantees that the peak temperature is reached before the plate is actively heated up to baseline temperature. Peak temperatures and ramp times different from the default ones should be used only after rigorous testing and with great care to avoid any injury or intolerable severe pain for participants.

At the beginning of each experiment, the temperature and timing values are uploaded to the ATEC temperature controller and the plate is controlled to the baseline temperature. After the adaptation phase, the temperature ramp starts. VAS pain ratings, hand-plate contact force, and actual plate temperatures are recorded every two seconds during the adaptation, ramping, and hold phases.

Relevant data are saved in csv format. The output file name includes the participant and session numbers. Absolute times are included for all rows (i.e., in two-seconds-intervals), which facilitates subsequent linking of temperatures, hand-plate contact force, and VAS ratings with other assessment-derived measures using external devices, such as timestamped ECG. The add-on program, plot_CAREST, lets users insert output files via a drag-and-drop feature and plots contact force and VAS pain ratings vs. temperature, as well as ratings for the hypothetical vignettes, facilitating a quick check of data recorded during an individual experiment. Before first use, we record a complete experimental run to confirm the correct configuration parameters and that the hardware works properly.

## Pilot study

### Participants and design

We tested the usability of CAREST and the feasibility of the setup using heat and cold pain experiments with 94 participants. We recruited the participants from university, clinical staff, and general population contexts (e.g., by presenting the study at a public event). They were 27.6 years old on average (SD = 8.4); 53 were female, 41 male. Assessments lasted approximately 45–50 min overall, including heat- and cold-based pain experiments in randomized order and a ~ 20–25-min break between the two tests. Before and after each test, participants completed a set of socio-demographic and psychological questionnaires, which we will associate with pain sensation and behavior in future work. Participants received monetary compensation (30 CHF); upon request, university students could instead receive ECTS credits for research participation hours.

All participants provided written informed consent prior to the assessment. The cantonal ethics committee Bern (BASEC#2023–01035) approved the study, and we conducted it in accordance with the ethical standards set out in the 1964 Declaration of Helsinki and its later amendments.

### Statistical analysis

To generate insights into the usability of the data produced by our tool, along with average values across key pain measures in an adult sample, we computed descriptive statistics for a selection of measures described in Table 1 for the heat and cold experiments. First, we focused on established measures (i.e., temperature at pain threshold and tolerance; pain intensity at pain threshold, noxious temperatures, and pain tolerance). Second, we zoomed in on measures related to hand-plate contact force (i.e., sample distributions for the baseline phase during the experiment, pain threshold, noxious temperatures, and pain tolerance; correlations with other measures; approach versus avoidance indicators). The statistical analyses were conducted using R (version 4.4.1) and RStudio (Posit Team, 2024).

## Results

We obtained quantitative data from almost all participants; in only one case, plate temperatures were not saved in the data file because the USB connection between the plate and laptop was interrupted. The mean self-ratings of pain intensities associated with three hypothetically painful situations, “falling into soft snow” (mean ± SD: 19 ± 18), “scrape knee” (54 ± 18), and “pinch fingers” (82 ± 12), show that the participants clearly differentiate between the different levels of pain and used the full range of the VAS scale. Trajectories of the VAS pain rating and hand-contact force versus temperature for heat and cold pain experiments of all participants are depicted in Figs S1–S4.

**Fig. 4:**
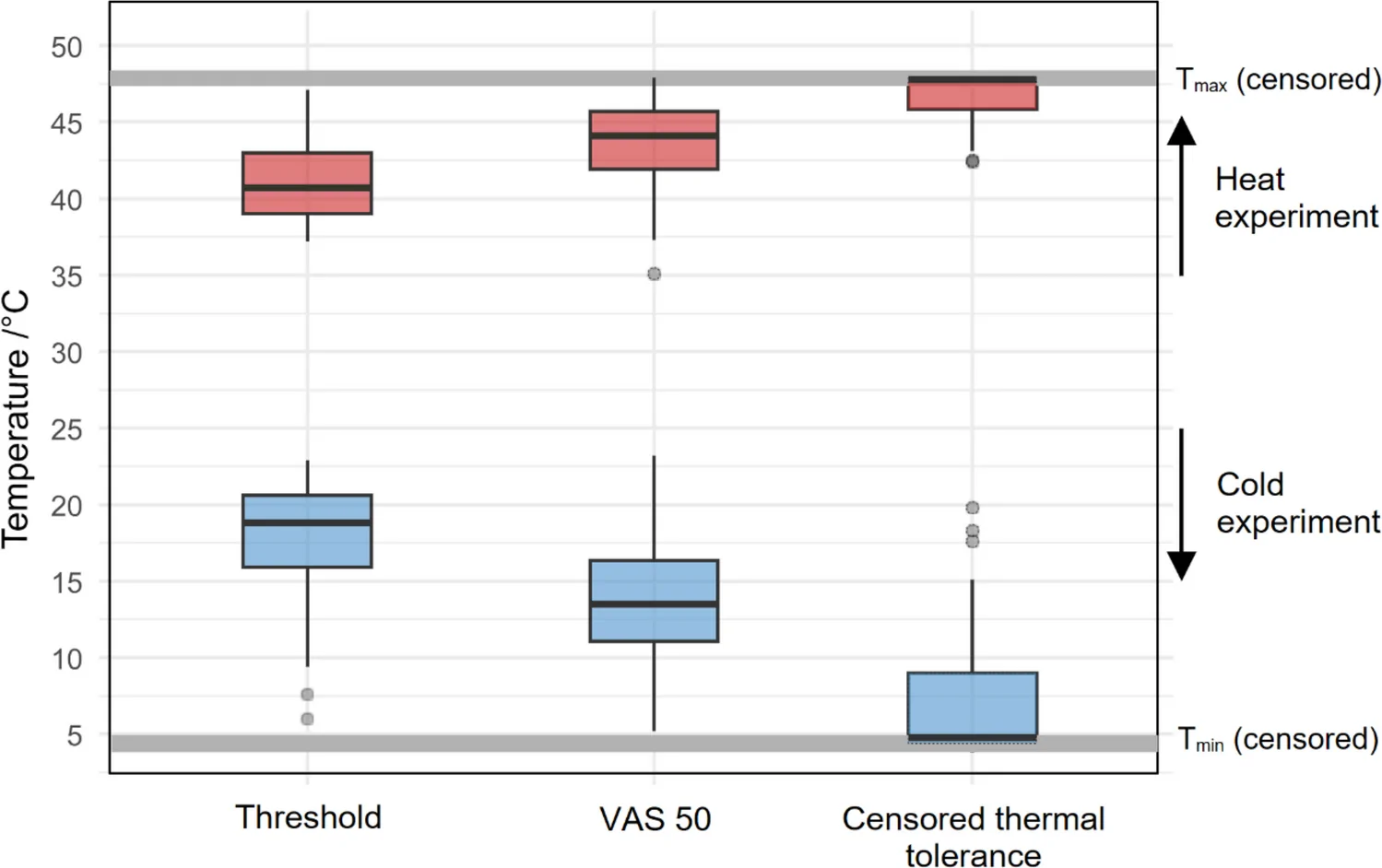
Temperatures at threshold (*n* = 93), censored tolerance (*n* = 94), and at VAS pain ratings of 50 (cold: *n* = 80, heat: *n* = 81) for heat (*red*) and cold (*blue*) pain experiments

**Fig. 5:**
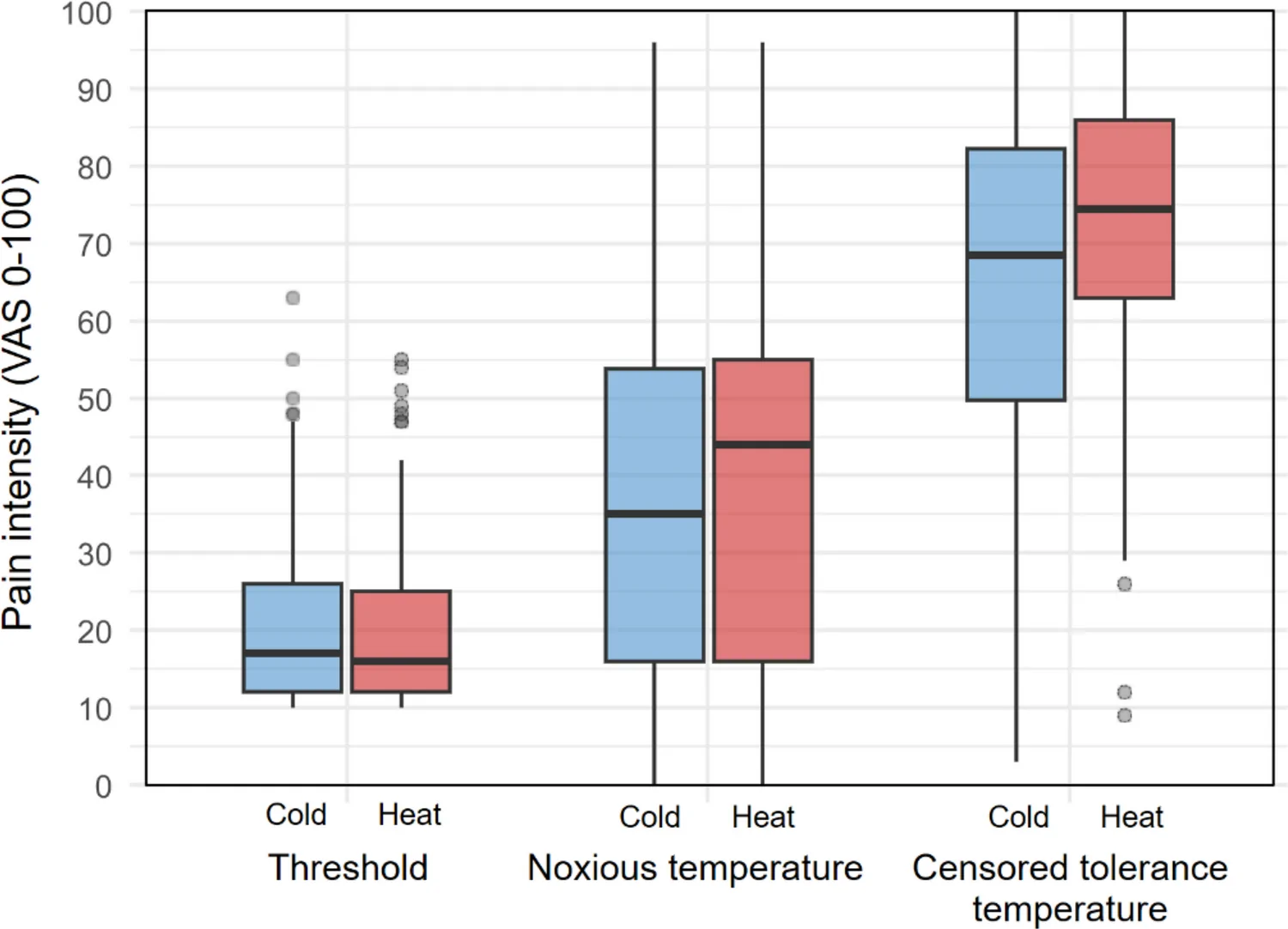
Pain intensity ratings (VAS) at threshold (*n* = 93), noxious (15 °C, cold: *n* = 90; 44 °C, heat: *n* = 84), and censored tolerance temperatures (cold: *n* = 92, heat: *n* = 94) for heat (*red*) and cold (*blue*) pain experiments

Statistical analyses of the heat pain experiments (Fig. 4) revealed pain threshold (mean ± SD: 41.1 ± 2.6 °C) and censored tolerance temperatures (46.8 ± 1.6 °C) in agreement with values reported earlier for healthy adolescents (41.8 ± 3.4 °C and 46.7 ± 2.2 °C, respectively (van der Venne et al., 2021)), where authors used a similar plate and an identical heating rate, but participants were advised only to start VAS pain rating upon their first pain sensation. In our experiments, we defined pain threshold as the temperature of a first significant increase of the VAS pain rating (two standard deviations + 10) from the VAS baseline calculated in the temperature interval between 36 °C and 37 °C for heat and between 23 °C and 24 °C for cold (see section Measures derived from CAREST data). Threshold and censored tolerance temperatures derived from the cold pain experiments were 18.0 ± 3.6 °C and 6.8 ± 3.7 °C. For both experiments, the mean VAS pain rating increased significantly from threshold (VAS_heat_ = 20.7 ± 11.8, VAS_cold_ = 20.7 ± 12.2) to tolerance (VAS_heat_ = 72.0 ± 20.0, VAS_cold_ = 65.6 ± 21.4) (Fig. 5).

Tolerance distributions are asymmetric (see Fig. 4) and thus censored tolerance values may be underestimated because of the limitation that the maximum and minimum temperatures were set to 47 °C and 5 °C, respectively. The experiments were automatically terminated for 56.4% (heat pain) and 61.7% (cold pain) of the participants for safety reasons. This is a general limitation in determining tolerance values (Park et al., 2019; van den Bosch, van Dijk, Tibboel, & Valkenburg, 2017). However, our time-synchronous recording of temperatures and VAS ratings may facilitate computation of additional characteristic measures that could be derivations of an estimate of pain tolerance, e.g., the temperatures at which VAS ratings exceed mild pain (VAS = 50) (Fig. 4) or the VAS rating at noxious temperatures (Fig. 5). Mean temperatures for mild pain (heat: 43.3 ± 3.0 °C, cold: 13.6 ± 4.3 °C) in our sample are within the range of the respective noxious temperatures (Dubin & Patapoutian, 2010).

The variability of our temperature data, as reflected in the VAS trajectories (S1, S2) and by the spread of data shown in Fig. 4 (0th–100th percentile), is consistent with those reported previously, e.g., QST reference data from the German Research Network on Neuropathic Pain (DFNS) (Rolke et al., 2006). For heat pain and cold pain thresholds examined on the hand of a cohort of 180 healthy adults the authors reported that the reference interval (2.5th to 97.5th percentiles) ranges from about 37–50 °C (heat pain threshold) and 29–0 °C (cold pain threshold).

VAS pain ratings (Fig. 5) did not differ between cold and heat conditions at the pain threshold. However, at higher intensity levels, VAS ratings were lower in cold conditions compared to heat conditions. This difference was statistically significant in the noxious temperature range (Wilcoxon test, *p* = 0.034) and especially at censored tolerance temperatures (*t* test, *p* < 0.001; Wilcoxon test, *p* < 0.001). No significant differences in distribution width were observed between conditions (see also Fig. 5).

**Fig. 6:**
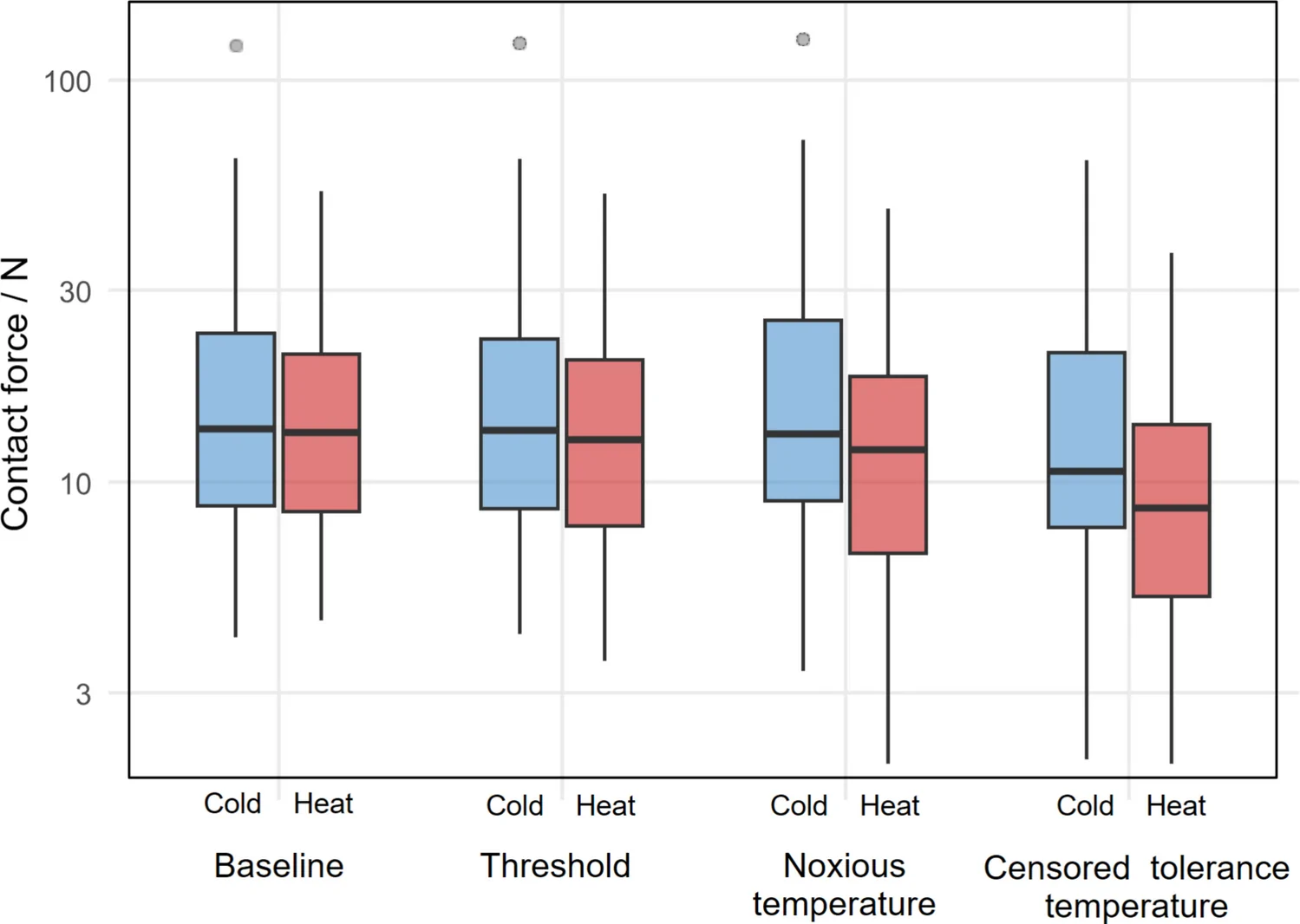
Contact force at baseline (cold: *n* = 91, heat: *n* = 93), threshold (cold: *n* = 90, heat: *n* = 92), noxious (15 °C, cold: *n* = 87; 44 °C, heat: *n* = 84) and censored tolerance temperatures (cold: *n* = 91, heat: 93) determined from heat (*red*) and cold (*blue*) pain experiments

In the present study, we did not feed back the hand-plate contact force to the participant. Instead, we monitored changes in force as a measure of participant behavior to observe approach to, or avoidance of, pain. Individual mean hand contact force values typically ranged from 2.7 N to 40.9 N, with one outlier (109.7 N) (Fig. 6). The median force was consistent with predominantly passive hand placement on the plate surface, whereas the force observed in the outlier would require active downward pressure comparable to leaning on the hand. The linear correlations between contact force and selected pain measures were weak to negligible (Table 3). Thus, in the present sample and within the range of hand-plate contact force observed in this study, the effect size of hand contact force on these measures is small, with limited explanatory power or no average effect. Future experiments with controlled hand-plate contact force, expanding the range below 2 N, will further examine a potential impact of contact force on pain measures.

**Table 3:** Linear correlation coefficients, *r*, between hand-plate contact force (averaged between threshold and tolerance temperatures) and different pain measures

	Temperature at…		VAS pain rating at…	
	pain threshold	final data point	pain threshold	final data point
Heat pain experiment	0.09 (*p* = 0.411)	0.02 (*p* = 0.884)	– 0.17 (*p* = 0.115)	0.18 (*p* = 0.080)
Cold pain experiment	0.03 (*p* = 0.778)	– 0.09 (*p* = 0.401)	– 0.15 (*p* = 0.147)	0.04 (*p* = 0.736)

**Fig. 7:**
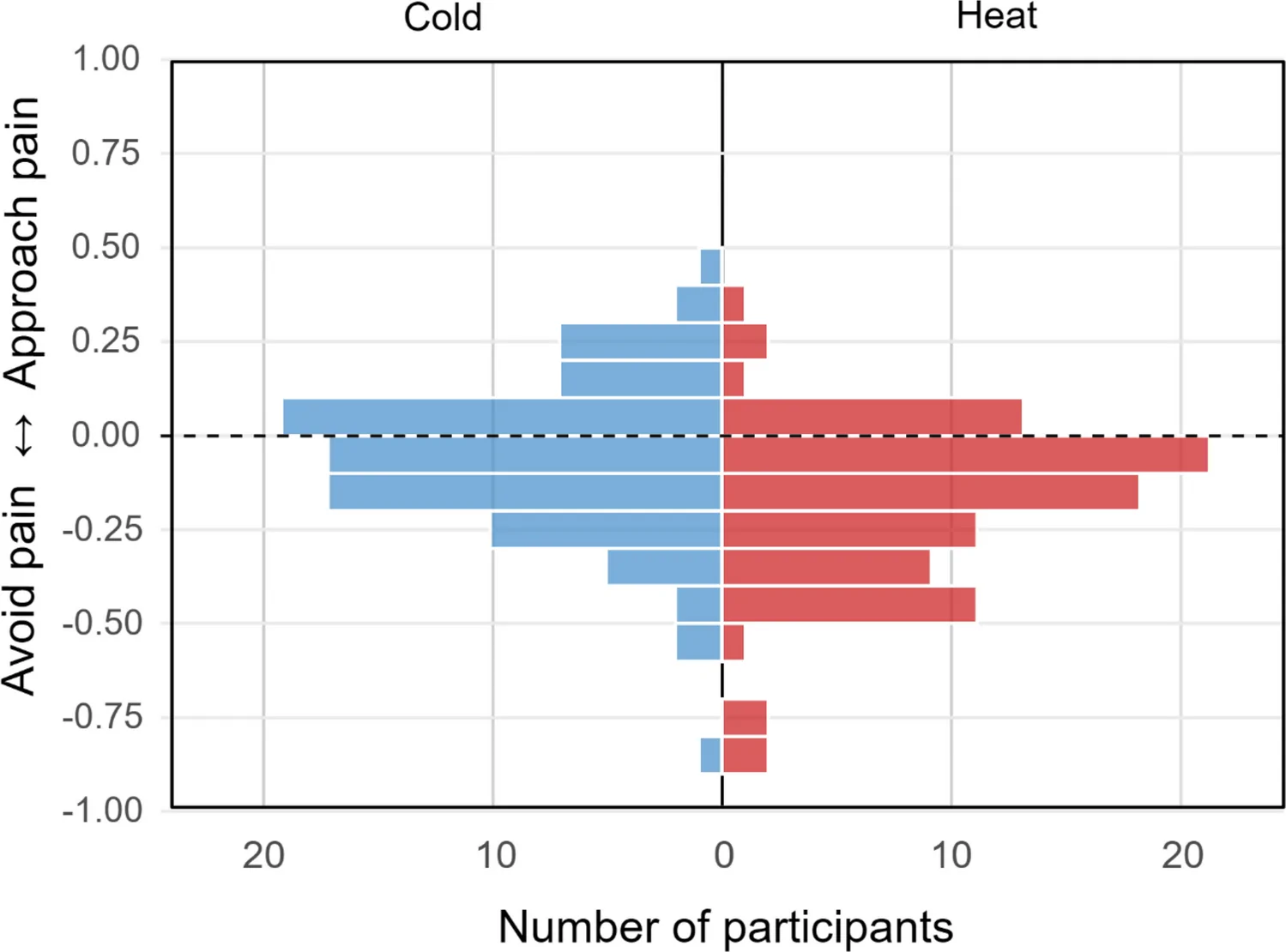
Individual behavioral tendency of approaching (positive values) or avoiding (negative values) cold (blue, *n* = 90) or heat (red, *n* = 92) pain. The ordinate shows the average contact force in the temperature interval between threshold and (censored) tolerance, minus the average contact force recorded between the start of the experiment and threshold, divided by the contact force averaged over the entire range. The plot reveals a modest tendency to avoid pain (mean ± SD, cold: – 0.06 ± 0.21, heat: – 0.18 ± 0.22); individual responses range from 0.49 to – 0.87

We observed modest but significant changes in contact force during the pain experiments when comparing median values at baseline and tolerance temperatures (Wilcoxon signed-rank test; cold: *p* = 0.03; heat: *p* < 0.001). These changes are more pronounced for the heat pain experiments (paired *t* test, *p* = 0.001; Wilcoxon signed-rank test, *p* < 0.001). Thus, not only are VAS pain ratings at noxious and tolerance temperatures for heat pain higher than for cold pain (Fig. 5), but also the median behavioral tendency to avoid this situation is increased for heat compared to cold pain experiments (Fig. 6). In all cases, the recorded contact force was larger than 2 N, documenting a stable contact area between the subject’s hand and plate surface. To uncover the individual tendency to avoid or approach pain, we calculated the individual difference of average contact forces in the intervals below and above pain thresholds relative to the overall contact force during the experiment (Fig. 7). The values for these changes reveal that, on average, hand contact force decreased by 6% (cold) and 18% (heat) in the interval between pain threshold and tolerance compared to the interval before reaching one’s pain threshold. The wide range from 0.49 (pain approach) to -0.87 (pain avoidance) highlights substantial interindividual heterogeneity, with one out of five participants differing markedly from the modest average tendency toward pain avoidance.

## Discussion

Temperature sensitivity, pain processing, and pain behavior appear to play an important role in psychopathology across the lifespan (Kao et al., 2024; Myers et al., 2006; Pontén et al., 2025; Schmahl & Baumgärtner, 2015). However, experimental assessments of pain sensitivity, especially with young participants, present unique ethical and methodological challenges. Experimental setups should be well tolerated, easily manageable and applicable to children as well as to adults. Furthermore, tools are required to provide reliable and reproducible data. CAREST facilitates a high level of standardization by controlling and logging assessments of temperature and pain sensitivity via thermal stimulation of the participant’s hand using a cold/hot plate and by recording hand-plate contact force trajectories.

Prior research has successfully employed hot plates with adolescent participants (Kao et al., 2024; van der Venne et al., 2021). The tool presented here builds on and advances this methodology by synchronously recording VAS ratings, plate surface temperature, and hand-plate contact force. It thereby facilitates the computation of several established and novel measures of distinct components of pain processing within a single experimental setting, while minimizing measurement error by (a) enabling standardized hand-plate contact force—or allowing researchers to account for interindividual differences therein, (b) continuously logging real-time data, and (c) providing exact data on both the timing of participant-initiated trial termination (i.e., when hand-plate contact force reaches zero) and the corresponding pain intensity ratings.

The setup described here can be adapted to specific research purposes by adjusting the preprogrammed temperature ramp and customizing the background image displayed on the touch screen for self-ratings. For example, to mimic the widely used cold pressor task (Birnie, Parker, & Chambers, 2016), researchers may configure CAREST to pre-cool the plate to, for example, 5 °C and maintain that temperature during the self-rating phase. Alternatively, researchers can conduct two experimental runs: the first to determine an individual participant’s pain threshold, and the second to maintain the temperature at this threshold (or, for example, at a moderate pain level) to assess endurance to a sustained stimulus. Such a configuration may also be suitable to detect sensitization or habituation effects (e.g., when pain intensity ratings change over time, although the temperature is held constant) (Potvin et al., 2024).

Ramps with increasing or decreasing temperatures, as used in our pilot study, can be varied to disentangle the roles that stimulus temperature and exposure time may play in pain threshold (Helme et al., 2004), pain tolerance, and endurance. Finally, researchers can create their own images (e.g., including different emoticons, numbers, or text) to re-scale the VAS displayed as background of the touch screen slider, to employ alternative rating scales. For example, while the self-rating scale used in our study ranged from “no pain” to “very severe pain,” other common scales range up to “worst pain imaginable” (Nielsen et al., 2009).

The rich data set produced by CAREST may enable the derivation of conversion or correction factors, allowing for more nuanced interpretation of results, especially in research that relies exclusively on self-report rather than experimental data. For example, comparisons between self-ratings from hypothetical pain scenarios and ratings obtained during experimental stimulus exposure may help validate and contextualize self-report data in studies that cannot include actual pain induction.

Because thermal transfer depends on the contact area between the skin and the plate, uninterrupted, uniform stimulation throughout the experiment may be important (Bakkers et al., 2013). Although experimenter instructions typically require participants to maintain firm hand contact with the plate until the pain becomes intolerable (van der Venne et al., 2021), the actual quality and consistency of this contact often could not be directly monitored. Additionally, factors such as social desirability may affect data quality (Logan, Claar, & Scharff, 2008); for example, if participants try to demonstrate high pain endurance and—consciously or unconsciously—lower the force of hand contact to reduce the hand–plate interaction area to endure longer. CAREST provides a novel approach to address this methodological gap by accurately monitoring the contact quality. The small correlation coefficients between participants’ average hand contact force across the experiment and other pain measures (e.g., pain threshold) suggest that, within the range of hand-plate contact force observed in our present study, hand contact force is not systematically linked with pain processing. In the future, we will investigate whether a systematic impact of hand contact force is observable within lower ranges of this measure, which may be relevant especially when conducting research with diverse populations in terms of body height and weight (e.g., when including and comparing children and adults). Moreover, counteracting factors within the sample may have partially masked a stronger underlying association (e.g., higher levels of hand contact force being associated with lower pain tolerance on the within-person level and with higher pain tolerance on the between-person level), which will also be a subject of future research.

If needed to enhance data reliability and interpretability, feeding hand-plate contact force values back to the participant during the experiment enables self-regulation towards standardized values or conscious modulation of perception. Such a setup also extends CAREST’s potential applicability to clinical intervention practices (e.g., to modulate a patient’s perception and interpretation of certain somatic sensations). Furthermore, the tool can be used to explore participant or situational characteristics that are associated with the tendency to manipulate experimental outcomes (e.g., to enhance one’s perceived endurance). CAREST thus contributes to a line of research that investigates the mechanisms underlying biased participant reporting through a triangulation of biological, behavioral, and self-report data (Steinhoff et al., 2023).

In the present study, we conceptualized and implemented the assessment of hand contact force as an indicator of physiological tension or behavioral approach-avoidance tendencies in response to thermal stimuli and pain sensation, and anticipation thereof. While most participants showed a modest tendency toward pain avoidance, one out of five exerted greater force upon reaching their pain threshold. Hand-plate contact force may thus, indeed, be used to assess physiological reactions and behavioral components relevant to the associations between pain physiology, pain processing, and psychosocial functioning. In the future, we will examine how the behavioral factors assessed based on hand contact force, along with other aspects of pain processing, are associated with psychopathology, an inclination toward self-harm and risk-taking, and, ultimately, overall health. Importantly, future research is also needed to clarify the extent to which contact force dynamics reflect avoidance and approach, how these are related to psychological traits, such as anxiety, and to what extent contact force dynamics may reflect other factors, such as muscular tension, fatigue, or attempts to maintain stable contact for the sake of instruction compliance.

Our pilot study aimed to test our standardized protocol and to provide reference values from a group of young adults. A single heat or cold pain experiment is easy to conduct, can be completed in less than 15 min, and may therefore also be useful in clinical practice for diagnostic purposes, for example, if certain parameters emerge as strong correlates of specific clinical conditions.

The conscious discrimination between thermal discomfort and thermal pain and its translation into a VAS scale may be challenging, especially for younger participants who may have less experience with diverse noxious stimuli. Furthermore, individual differences in the interpretation of qualitative anchors can introduce variability in VAS ratings. To mitigate these effects, participants completed a familiarization process using hypothetical scenarios of increasing imaginary pain intensity, ensuring a consistent understanding of the scale’s directional logic (i.e., that increasing intensity requires higher numerical ratings). Because synchronous recordings of VAS ratings and hot/cold plate temperatures allow us to track participants' reactions to the perceptual onset of pain, we propose identifying the pain threshold as the significant increase in VAS ratings from baseline to minimize individual differences in anchor or VAS scale interpretation. Because the perceptual onset of pain precedes its cognitive labeling, the latter may emerge at higher temperatures for heat pain or lower temperatures for cold pain, as participants may require a higher degree of certainty before categorizing a sensation as ‘pain’ (Kuhtz-Buschbeck et al., 2010). This behavior is reflected in Fig. 4, where the median threshold temperature and the temperatures at which ‘mild pain’ (VAS 50) were reached are clearly separated.

When comparing our pain threshold and tolerance values with those published in the literature, the comparably low rate of temperature change and large stimulated skin area must be considered. Low rates of temperature change and spatial summation effects reliably lower the heat pain threshold (Bakkers et al., 2013; Park et al., 2019; Rasmussen et al., 2015) and lead to significantly higher pain ratings (Vierck et al., 2013). Our pain threshold and tolerance temperatures for the heat pain experiment are in agreement with those reported for healthy adolescents as published by van der Venne et al. (2021), who used a similar plate and an identical heating rate, providing strong evidence for the experiment’s reproducibility. Heat pain threshold temperatures for the hand and palm were 2.5 and 1.9 °C higher, respectively, in the study of Park et al. (2019), who used a *s*imilar heating rate (0.1 °C/s*)* but a much smaller thermode area (1 cm^2^). Comparison of our cold pain threshold and tolerance values with published data is difficult because cold pain experiments performed with similar cooling rates and thermode contact areas are not available. Pain thresholds determined with higher cooling rates (1 °C/s) and smaller thermode area (9 cm^2^) for the dorsum of the hand were reported to be in the range between 15.3 and 17.6 °C (Wasner & Brock, 2008), which is comparable to our results for the palm (18.1 °C)*.* The results from our present study may serve as reference values for future studies with the present setup.

## Conclusion

CAREST is a flexible tool tailored for experiments with children, adolescents, and adults, facilitating comparability of pain processing data across the lifespan. The tool generates quantitative data on thermal and pain sensitivity in a simple way, using a heat/cold plate placed on electronic load cells and connected to a single touch screen device. Real-time recordings of temperature, self-ratings of stimulus or pain perception, and hand-plate contact quality fill gaps in prior research. By providing continuous, high-resolution timestamped recordings, CAREST enables precise temporal alignment with concurrently acquired physiological signals (e.g., ECG, EEG), facilitating integrative analyses of neuronal, physiological, cognitive, and behavioral pain processing. Data collected using CAREST bear the potential to advance our understanding of how different measures and concepts of pain processing are related to each other, and to make results from pain research using thermal paradigms and heat/cold plates more comparable. CAREST may thus be useful for researchers interested in conditioned pain modulation, offset analgesia, the thermal grill illusion, and related sensory phenomena. Ultimately, the tool is intended to support research into the biological mechanisms underlying human psychosocial health by generating rich, multimodal data sets that integrate self-reports with objectively measured parameters, including hand contact force to monitor pain behavior.

## Supplementary Information

Below is the link to the electronic supplementary material.

Supplementary file1 (PDF 440 kb)

## Data Availability

The raw data from the pilot study are visualized in the supplementary figures. Electronic files of raw data are available from the corresponding author upon reasonable request, for those participants who provided consent for data sharing, and subject to applicable confidentiality regulations given that the data are linked to sensitive information. To illustrate the structure and content of the raw data files generated by CAREST, example data files generated by the authors (self-experiments) are available on GitHub in the folder https://github.com/hjuergensteinhoff/CAREST/tree/main/examples. Figure 3: 999992_001_CAREST.csv.

## Code availability

The source code is available on GitHub (https://github.com/hjuergensteinhoff/CAREST).
