# Supplementary material for: Real-time assessment of temperature sensitivity, pain processing, and pain behavior across the lifespan: Integrating stimulus data, contact force, and subjective ratings
Source: Behav Res Methods. 2026 Oct 14;58(11):321. doi: 10.3758/s13428-026-03192-w (PMC13669072; doi:10.3758/s13428-026-03192-w)

Real-Time Assessment of Temperature Sensitivity, Pain Processing and Pain Behavior Across the Lifespan: Integrating Stimulus Data, Contact Force, and Subjective Ratings

Heinz-Jürgen Steinhoff[1], Besim Prenaj[2,3], Sebastian L. Kübel[2], & Annekatrin Steinhoff[2]

[1]University of Osnabrück, Germany, [2]University Hospital of Child and Adolescent Psychiatry and Psychotherapy, University of Bern, Switzerland, [3]Graduate School of Health Sciences, University of Bern, Switzerland

Supplementary Figures 1 - 4

For Review Only

Supplementary Figure 1

Individual heat pain ratings by temperature

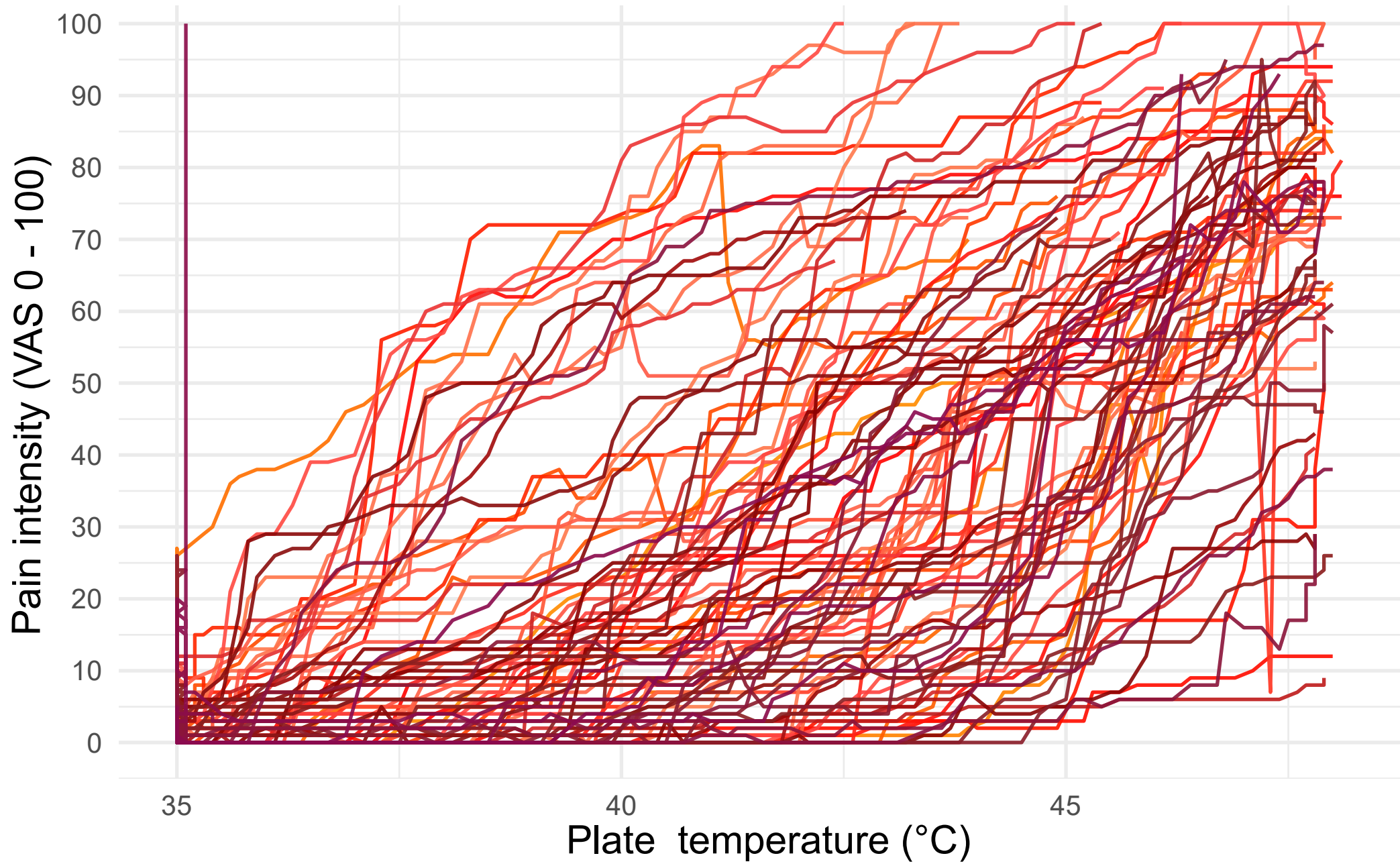

Supplementary Figure 2

Individual cold pain ratings by temperature

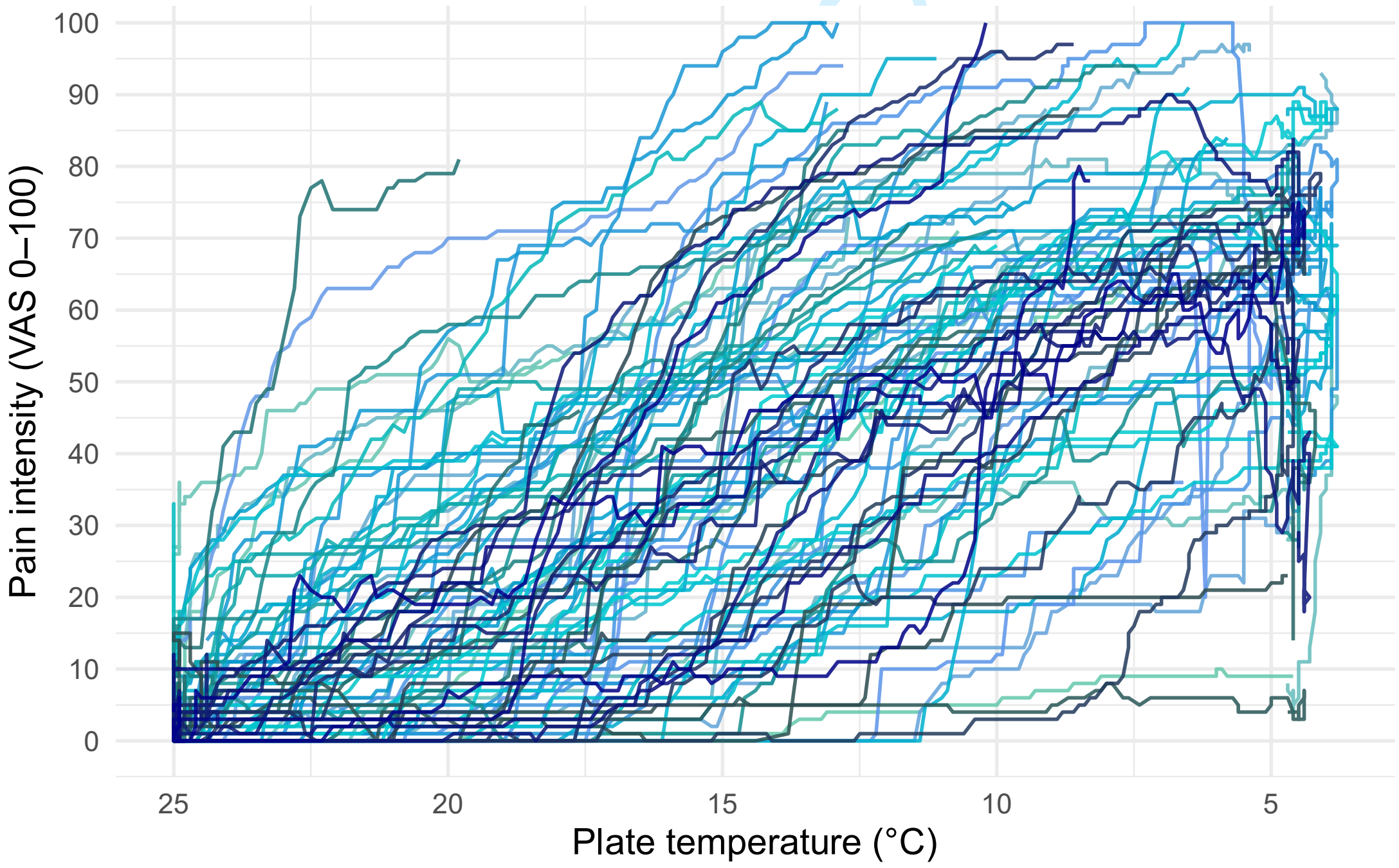

Supplementary Figure 3

Heat contact force by temperature

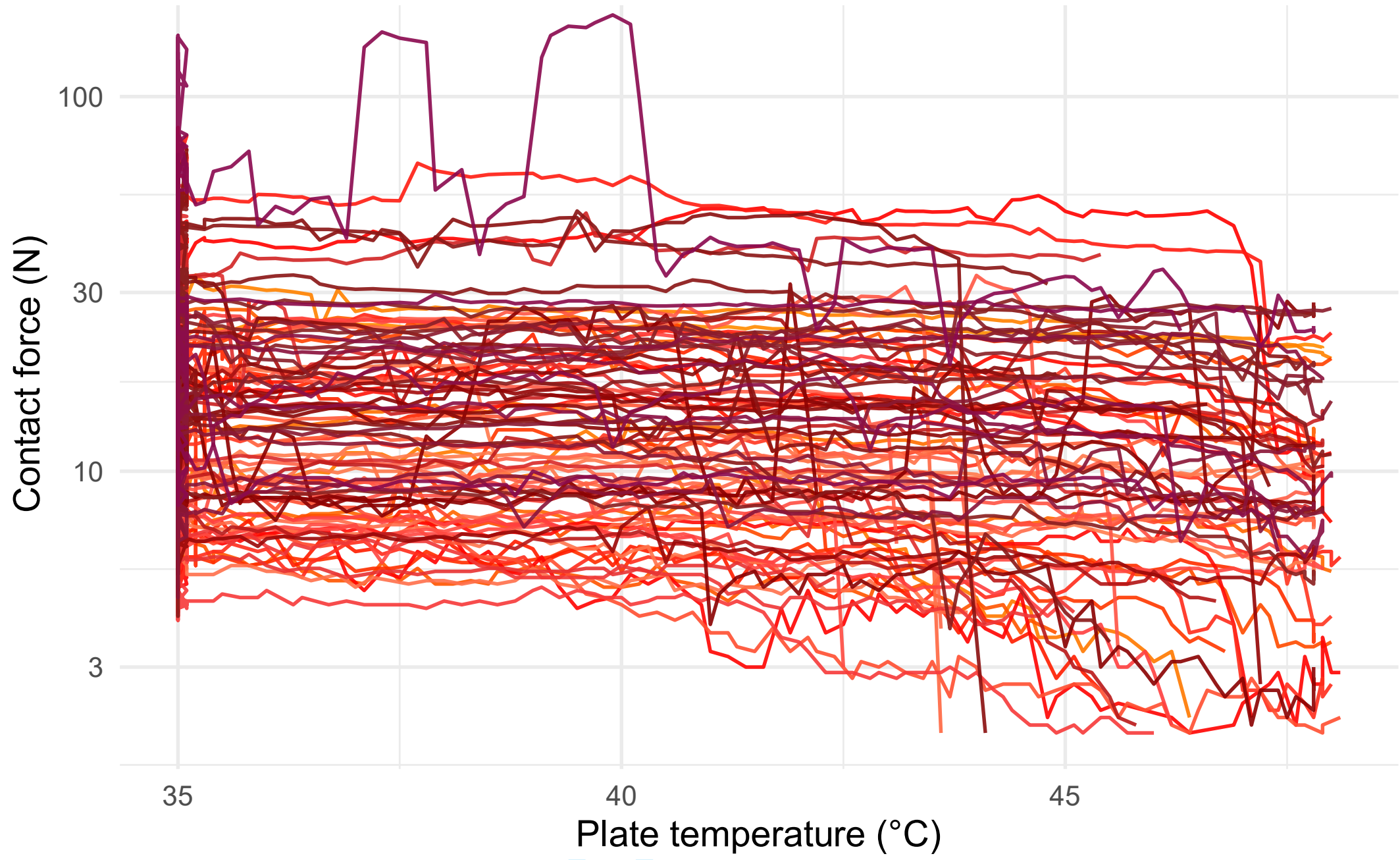

Supplementary Figure 4

Cold contact force by temperature

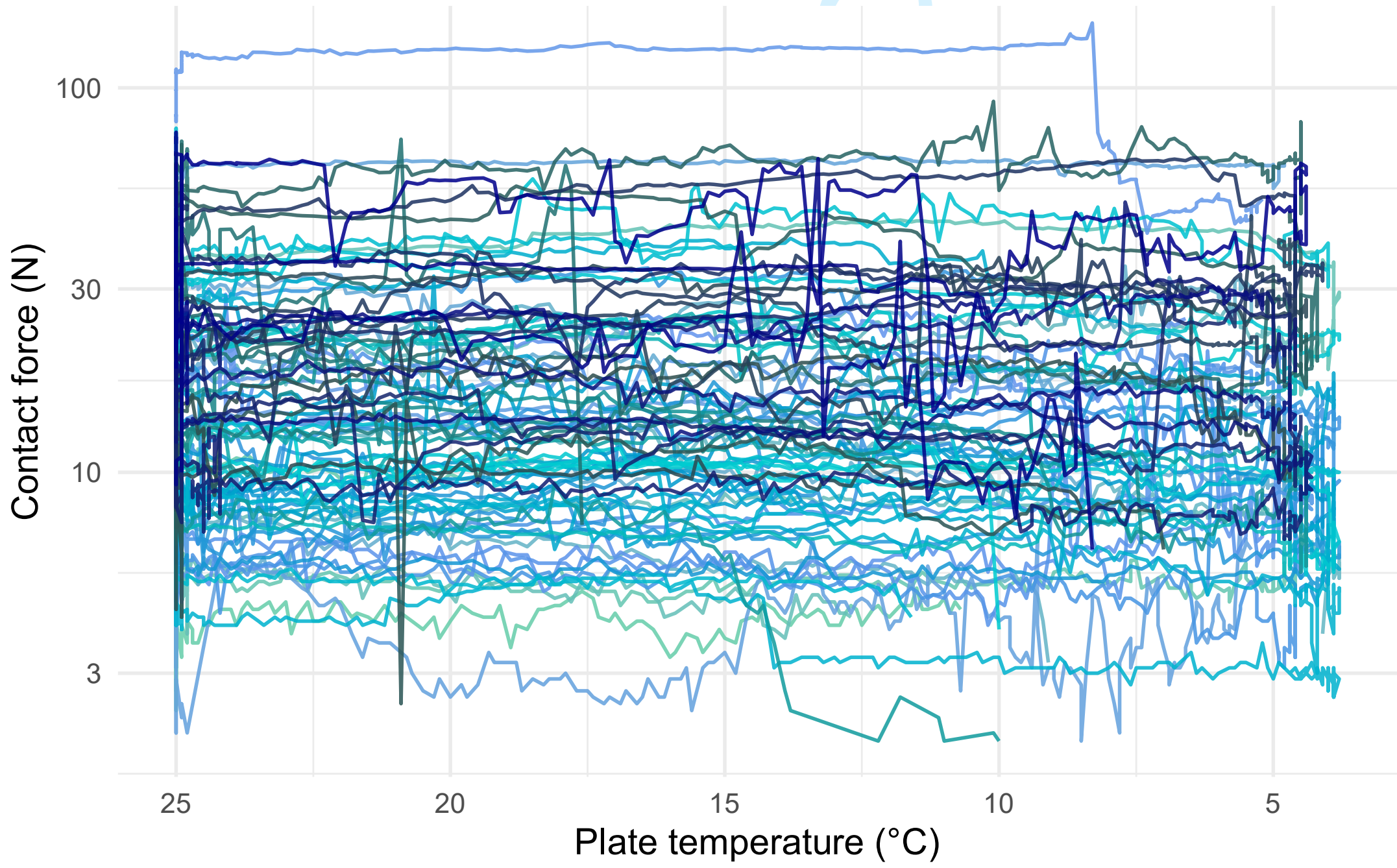

Supplement: Supplementary file 1 [file 13428_2026_3192_MOESM1_ESM.pdf]
